# Supplementary material for: Mass Spectrometry-Based Proteomic Analysis of Potential Host Proteins Interacting with N in PRRSV-Infected PAMs
Source: Int J Mol Sci. 2024 Jun 29;25(13):7219. doi: 10.3390/ijms25137219 (PMC11241482; doi:10.3390/ijms25137219)
Supplement: Supplementary file 1 [file ijms-25-07219-s001.zip › supplementary materials/Table S2.pdf]

Table S2. Primer sequences used in this study.

Name[1]	Sequences (5′ to 3′)[2]	GenBank	Product size
Flag-DDX1-F	AAGGACGACGATGAC*AAGCTT*ATGGCGGCCTTCTCCGAG	XM_021087822	2223bp
Flag-DDX1-R	CCTCGAGAGATCTCG*GTCGAC*TCAGAAGGTTCTGAACAGCTGGT		
Flag-DDX3X-F	AAGGACGACGATGAC*AAGCTT*ATGAGTCATGTGGCGGTGGA	NM_001246203	1986bp
Flag-DDX3X-R	CCTCGAGAGATCTCG*GTCGAC*TCAGTTGCCCCACCAGTCAA		
Flag-SAMHD1-F	AAGGACGACGATGAC*AAGCTT*ATGCAGAGTGCCGACTCCC	NM_001292105	1884bp
Flag-SAMHD1-R	CCTCGAGAGATCTCG*GTCGAC*TCACACCGAGTCCTTTGCAAA		
Flag-PCBP2-F	AAGGACGACGATGAC*AAGCTT*ATGGACACCGGTGTGATTGAA	NM_001244512	1062bp
Flag-PCBP2-R	CCTCGAGAGATCTCG*GTCGAC*CTAGCTGCTCCCCATGCCA		
Flag-EIF3G-F	AAGGACGACGATGAC*AAGCTT*ATGCCTACCGGAGACTTTGATTC	XM_021083783	963bp
Flag-EIF3G-R	CCTCGAGAGATCTCG*GTCGAC*TTAGTTGGTTGACGGCTTGGC		
Myc-N-F	CCC*AAGCTT*ATGCCTAATAACAACGGCAAGC	KX766378	372bp
Myc-N-R	CCG*CTCGAG*TCATGCTGAGGGTGAAGCTG		
PCBP2-qF	CTGCGTGGTCATGTTGGA	NM_001244512	118bp
PCBP2-qR	GTCGCTGCCTGTGCTGTA		
β-Actin-qF	CGGGACATCAAGGAGAAGC	EU655628	132bp
β-Actin-qR	CTCGTTCCCGATGGTGATG		

[1] F: forward primer, R: reverse primer, q: The primers were used for RT-qPCR.

[2] The italicized alphabets indicate restriction enzyme cleavage sites for cloning.
